# Accuracy of mutual predictions of plant and microbial communities vary along a successional gradient in an alpine glacier forefield

**DOI:** 10.3389/fpls.2022.1017847

**Published:** 2023-01-13

**Authors:** Xie He, Maximilian Hanusch, Victoria Ruiz-Hernández, Robert R. Junker

**Affiliations:** ^1^ Department of Environment and Biodiversity, Paris Lodron University of Salzburg, Salzburg, Austria; ^2^ Evolutionary Ecology of Plants, Department of Biology, Philipps University of Marburg, Marburg, Germany

**Keywords:** bacteria, environment, fungi, plants, predictive models, succession

## Abstract

Receding glaciers create virtually uninhabited substrates waiting for initial colonization of bacteria, fungi and plants. These glacier forefields serve as an ideal ecosystem for studying transformations in community composition and diversity over time and the interactions between taxonomic groups in a dynamic landscape. In this study, we investigated the relationships between the composition and diversity of bacteria, fungi, and plant communities as well as environmental factors along a successional gradient. We used random forest analysis assessing how well the composition and diversity of taxonomic groups and environmental factors mutually predict each other. We did not identify a single best indicator for all taxonomic and environmental properties, but found specific predictors to be most accurate for each taxon and environmental factor. The accuracy of prediction varied considerably along the successional gradient, highlighting the dynamic environmental conditions along the successional gradient that may also affect biotic interactions across taxa. This was also reflected by the high accuracy of predictions of plot age by all taxa. Next to plot age, our results indicate a strong importance of pH and temperature in structuring microbial and plant community composition. In addition, taxonomic groups predicted the community composition of each other more accurately than environmental factors, which may either suggest that these groups similarly respond to other not measured environmental factors or that direct interactions between taxa shape the composition of their communities. In contrast, diversity of taxa was not well predicted, suggesting that community composition of one taxonomic group is not a strong driver of the diversity of another group. Our study provides insights into the successional development of multidiverse communities shaped by complex interactions between taxonomic groups and the environment.

## 1 Introduction

Local microclimatic conditions, soil properties as well as the tight interactions between plants and belowground microbes shape the communities in natural ecosystems (Zak et al., 2003; Mouhamadou et al., 2013; Navratilova et al., 2019; Harrison et al., 2020; Ohler et al., 2020). pH, temperature and soil chemical properties have been shown to affect the plant and microbial composition by defining the niches available in a given location (Darcy et al., 2018; Dastogeer et al., 2020; Harrison et al., 2020; Davison et al., 2021; Junker et al., 2021). Additionally, the interactions between bacteria, fungi and plants strongly affect local communities. The interactions between plants and microbes, for instance, are mediated through plant root exudates and litter input that offer the carbon sources and provide various niches for microbes (Knelman et al., 2012; Lopez-Angulo et al., 2020). Likewise, microbes decompose the carbon and affect plants through the supply of available soil nutrients to plants such as nitrogen fixation (Schmidt et al., 2008; van der Putten et al., 2013) and the interplay of mutualistic and antagonistic effects determine if they will maintain plant community diversity or cause community convergence (Wardle et al., 2004; Bever et al., 2012; van der Putten et al., 2013; Teste et al., 2017; van der Putten, 2017). The outcome of pairwise interactions between bacterial, fungal and plant species is highly context dependent and may be modulated by the presence of other taxa as well as environmental conditions (David et al., 2020; Raza et al., 2020). For instance, environmental conditions such as temperature and soil moisture affect plant and microbes and can regulate plant-microbe associations (Rasmussen et al., 2019; Rudgers et al., 2020; Robroek et al., 2021), and increasing environmental stress alters microbial facilitation of plant germination or biomass production (David et al., 2020). This may complicate predictions on the composition and diversity of communities based on the knowledge of other taxa or environmental factors in natural ecosystems where environmental conditions strongly vary and thus may modulate the interactions between taxonomic groups. Nonetheless, the interdependencies between plants, bacteria and fungi may leave a signal in community composition and diversity within the taxonomic groups and thus these properties may be mutually predictable (Horn et al., 2017; Leff et al., 2018).

Successional gradients with considerable variation in soil properties and climatic conditions are an ideal study system to reveal how the interdependences between taxonomic groups change along a temporal and environmental gradient (Cannone et al., 2008; Walker et al., 2010; Chang & HilleRisLambers, 2016). Glacier forefields are prime examples of primary successions and for studies on the assembly of multidiverse communities (Ficetola et al., 2021; Hanusch et al., 2022). Receding glaciers provides barren substrates waiting for the successive colonization of organisms such as plants and soil biota (Bernasconi et al., 2011; Burga et al., 2010; Ficetola et al., 2021). The time for space substitution of chronosequences allows to track soil development and the processes that shape biotic communities (Walker et al., 2010; Chang & HilleRisLambers, 2016). Multiple studies described the successional trajectories of plant, bacteria and fungi communities and the development of soil conditions along glacier forefields (Bernasconi et al., 2011; Zumsteg et al., 2012; Buma et al., 2017; Buma et al., 2019; De Vries et al., 2021). However, these studies usually focus on one or rarely two specific taxonomic groups (Ficetola et al., 2021), thus a full consideration of multidiverse communities and environmental conditions may facilitate a general evaluation of associations between different biotic and abiotic parts. In this context, we applied random forest analysis to evaluate the interdependences between taxonomic groups and with environmental conditions to reveal the strength of mutual influences along a successional gradient. Machine learning algorithms have been increasingly applied for predictions using complex ecological data. For instance, random forest analysis has been used to explore the links between soil bacterial community composition and environmental factors (Hermans et al., 2020) and in predictions of species interactions based on traits for understanding interaction networks (Pichler et al., 2019). The high performance of machine learning algorithms and especially random forest is obtained by their ability to model non-linear combinations of numerical and categorial data without complex transformations resulting in estimates of the accuracy of predictions (Breiman, 2001; Ghannam & Techtmann, 2021; Goodswen et al., 2021).

Empirical studies have shown that plants and abiotic factors affect microbial communities at the same time but in different ways: they explain different parts of variation in soil microbial communities and different studies have shown contrasting results with either plants or environmental factors being more important in shaping microbial communities (Mitchell et al., 2011; Kruger et al., 2017; Leff et al., 2018; Reese et al., 2018; Cheng et al., 2020). In addition, plant species composition, functional identity, Shannon and phylogenetic diversity have been reported to show different associations with microbial communities (Dassen et al., 2017; Chen et al., 2018; Leff et al., 2018) and the relationships may change at different successional stages (Porazinska et al., 2018; Hanusch et al., 2022). Along the successional gradient of Ödenwinkel glacier forefields in the Austrian Alps, we considered a range of taxonomic groups (bacteria, fungi and plants) and environmental variables (plot age, temperature, and soil pH) to determine how well bacteria, fungi, and plant communities as well as environmental conditions serve as indicator for the composition and diversity of other taxonomic groups as well as environmental factors at different successional stages. We used multivariate datasets on plant species, bacterial operational taxonomic units (OTU), and fungi OTU composition as well as several environmental factors as explanatory variables. These variables were used to predict univariate data informing about the composition, functional composition and Shannon diversity of plant, bacteria, fungi, plant phylogenetic diversity, plant functional diversity and environmental factors. We aim to address the following three questions: 1) Are bacterial, fungal, and plant communities as well as environmental conditions good predictors for the composition and diversity of the other taxonomic groups or environmental factors? 2) Is the accuracy of prediction variable along the successional gradient? 3) Are there single most important predictors for all taxonomic groups? Our study will reveal the relative importance of interactions across taxa and abiotic factors in shaping the diversity and composition of multidiverse communities along a successional gradient and will thus motivate future studies on the mechanisms underlying community assembly.

## 2 Materials and methods

### 2.1 Data collection

*Plots set up* - Our study site is located at the forefield of the Ödenwinkelkees glacier (Stubachtal valley, Hohe Tauern National Park, Austria; Dynamic Ecological Information Management System – site and dataset registry: https://deims.org/activity/fefd07db-2f16-46eb-8883-f10fbc9d13a3, last access: August 2021) (Junker et al., 2020). The Ödenwinkelkees glacier was covered by ice at the latest glacial maximum in the Little Ice Age (LIA; around 1850) but the glacier retreat released a transect of around 1.7 km long with the elevation between 2070 and 2170 m. In summer 2019 (26 June - 16 September), we established 135 permanent plots that were evenly distributed between the LIA glacier maximum and the current extent of the glacier (glacier tongue) within the glacier forefield, representing a chronosequence of succession with high temporal resolution. Each plot is a square with 1 m side length (resulting in an area of 1 m^2^) and a ground anchor is marking the center of the plot. Plot age was estimated according to its relative position compared with historical records of eight deglaciating periods (year 1850, 1890, 1929, 1969, 1977, 1985, 1998, 2013) (Junker et al., 2020).

*Plant survey and functional traits* - We identified all vascular plant species (*n* = 99) occurring at every plot and recorded the coverage of every species. We measured the plant height, leaf area, leaf weight and calculated the specific leaf area (SLA) for those 48 plant species that occurred in 10 or more plots. For three focus species we phenotyped up to three individuals on every plot where they occurred: *Oxyria digyna* as representative of early succession, *Trifolium badium* as representative of late succession, and *Campanula scheuchzeri* which occurred all along the successional gradient. For the other *n* = 45 species, up to five individuals per plot were phenotyped on the youngest, the oldest, and the intermediate plot where they occurred. Additionally, we obtained the functional traits of the plant species from Biolflor database (https://www.ufz.de/biolflor/index.jsp) for 92 species out of 99 plant species occurring in the field. We used nine functional traits which have been shown to be response traits to environmental changes at the community level (Kahmen & Poschlod, 2004; Bernhardt-Römermann et al., 2008; Aguiar et al., 2013; Hintze et al., 2013), including reproduction, diaspore type, leaf persistence, life form, life span, pollen vector, strategy type, type of reproduction, dispersal of diaspores.

*Soil bacteria and fungi sampling and sequencing* - We also characterized the soil microbiome (bacteria and fungi) of each of the plots. We sampled soil from each plot at two locations at a depth of 3cm, soil from two locations per plot were pooled to one sample for further analysis. We extracted microbial DNA from soil samples following the protocol of the ZymoBIOMICS DNA Miniprep Kit (Zymo Research, D4300). Microbiome analysis was performed by Eurofins Genomics (Ebersberg, Germany) using the company’s standard procedure. Sequencing was done using Illumina MiSeq and the sequenced regions were V3-V4 region of the 16S rRNA gene to identify bacterial OTUs and the ITS2 region for fungal OTUs following the standard procedure “InView - Microbiome Profiling 3.0 with MiSeq” (for detailed methods see Junker et al., 2020). Abundances of bacterial and fungal taxonomic units were normalized using lineage-specific copy numbers of the relevant marker genes to improve estimates (Angly et al., 2014). Prior to the random forest analysis of microbial communities, we performed a cumulative sum scaling (CSS) normalization (R package metagenomeSeq v1.28.2) on the count data to account for differences in sequencing depth among samples.

*Soil temperature and pH* - To record the seasonal mean temperature, we buried temperature loggers with a resolution of 0.5°C (MF1921G iButton, Fuchs Elektronik, Weinheim, Germany) 10 cm north of each plot center, at a depth of 3 cm below ground (Junker et al., 2020; Ohler et al., 2020) during field work in 2019. The thermo loggers were set to start on 13th August 2019 and were stopped on 9th August 2020 with a total of 2048 measurements recorded on 362 days. Seasonal mean temperature was calculated on the basis of the recordings between 13th August to 16th of September 2019 and 26th June to 9th August 2020 representing the period in which the plots were free of permanent snow cover before and after the winter 2019/2020. In 2020 (25 July - 21 August), we took additional soil samples from all plots to measure soil pH. Samples were sent to AGROLAB Agrar und Umwelt GmbH (Sarstedt, Germany) for analysis.

### 2.2 Data analysis

To test the predictability of the diversity and composition of each of the taxonomic group by the composition of other taxonomic groups as well as by environmental parameters, we used the machine learning algorithm random forest with R package *randomForest* (Liaw and Wiener, 2002). We used the 4 sets of variables as explanatory variables: community tables of plants, bacteria, and fungi with plots as rows and the abundance of the species or OTUs as columns (**Supplementary Table S1**, **S2**, **S3**), and environmental conditions of each plot with plots as rows and environmental variables as columns (**Supplementary Table S4**). As dependent variables we used univariate variables including the composition (principal components PCs), functional composition (PCs) and Shannon diversity of plant, bacteria, fungi, plant phylogenetic diversity, plant functional diversity as well as soil seasonal mean temperature, pH, plot age, resulting in 20 variables in total (**Supplementary Table S5**; **Supplementary Figure S1**, **S2**). Each set of explanatory variables were used separately to predict the variables related with other taxonomic groups or environmental factors. As random forest analysis can only deal with univariate dependent variables, we conducted the principal component analysis (PCA) using R package *vegan* (Oksanen et al., 2013) and used the first two PC axis which carry most information of the composition to refer to plant species composition (15.3% + 11.2%), bacteria composition (6.4% + 4.6%) and fungi composition (4.1% + 3.2%). Plant functional composition matrix includes the categorical functional traits obtained from BiolFlor database and the community weighted means of filed measured traits (plant height, leaf area, leaf weight and SLA) (**Supplementary Table S6**). For each category of each categorical trait, we calculated the total coverage of species belonging to the category, and this was done for all the 9 traits and all 9 traits were merged to a single table, thus generating the functional composition table with plots name as rows and 39 trait categories as columns, i.e. each categorial trait had two or more categories resulting in a total of 39 categories. Plant functional composition was represented by the first two PCAs, too (63.1% + 12.1%). Plant Shannon diversity was calculated from the compositional dataset using the R package *vegan*. Plant phylogenetic diversity was calculated using the R package *picante* (Kembel et al., 2010). We extracted a phylogenetic tree using the R package *pez* (Pearse et al., 2015) for species existing in our field site from a dated molecular phylogeny tree (32,223 species) for land plants (Zanne et al., 2014). In cases where species were not included in the tree, it was substituted by species from the same genus. Among 99 species existing in our plots, we were able to match and build a tree with 96 species and we used it for the calculation of phylogenetic diversity. We used ‘Functional dispersion’ calculated from the R package *FD* (Laliberte & Legendre, 2010) as the index for plant functional diversity. The BiolFlor traits and field measured traits of every species were used for the trait table identically for every plot, and for the community table the species with a low occurring frequency along the successional gradient (not included in the 48 species with traits measured) were ignored in the calculation of functional diversity. For bacteria and fungi, the Shannon diversity was calculated based on the OTU composition (without CSS normalization) after rarefying the data to the minimum number of reads (bacteria: 2117; fungi: 1420) available in the samples (repeats = 999). Additionally, we obtained bacteria functional composition represented by MetaCyc pathway abundances with PICRUSt2 (Langille et al., 2013), and fungi functional composition with FUNGuild to assign fungal OTU to different functional groups (Nguyen et al., 2016). Bacteria functional composition (**Supplementary Table S7**) was represented by the first two PCAs (78.0% + 7.2%), and fungi functional composition (**Supplementary Table S8**) was represented by the first two PCAs (33.2% + 17.0%), too.

Using all combinations of explanatory and dependent variables, we performed random forest analyses with 10-fold cross validations to quantify the performance of the predictive model, a total of 60 models. Specifically, for each prediction, 80% of the plots were randomly selected as the training dataset and the remaining 20% of the plots were used as test dataset. The predictive model resulting from the training dataset was applied to the test data and the predicted values of the plots in the test dataset were correlated with the observed values of these plots. This process was repeated for ten times, and then we defined the mean Pearson’s *r*-value of ten correlations as ‘accuracy of prediction’ and used the proportion of statistically significant correlations (p-value < 0.05) out of the 10 correlations as ‘significance frequency’. Additional to random forest analysis using all the plots for a global impression on the predictability of dependent variables, we also employed a moving frame approach to detect how the predictabilities change along the successional gradient. With the 135 plots, we grouped every 45 plots into one frame and used the median plot as identifier of the frame. Thus, the first frame included plots 1 to 45, the second 2 to 46, and so forth. This approach led to a set of 91 moving frames whose identifiers ranged from plot 23 to plot 113. Using the same proportion of training and test dataset, for every 45 plots in each frame, data of 36 (80%) randomly selected plots was used as training dataset, and the other 9 (20%) plots were used as test dataset. The accuracy of prediction and significance frequency were calculated for every frame as stated before. We fitted a linear or quadratic regression with the accuracy of prediction of every variable along the successional gradient as independent variable and the frame number as explanatory variable. The model with a higher *r^2^* value was chosen and the statistically significant relationships were shown as a regression line. We compared for each group how well they predicted every variable as well as for each variable how well they were predicted by every other taxonomic group or environmental factors along the successional gradient (except for the group that was considered in the dependent variable) using the Tukey Test. Note that our results do not imply a direction of effects in the sense that the dependent variable is affected by the explanatory variable. For instance, if bacterial communities statistically predict soil temperature it does not mean bacterial communities affect the soil temperature but rather are affected by this environmental parameter.

## 3 Results

**Figure 1:**
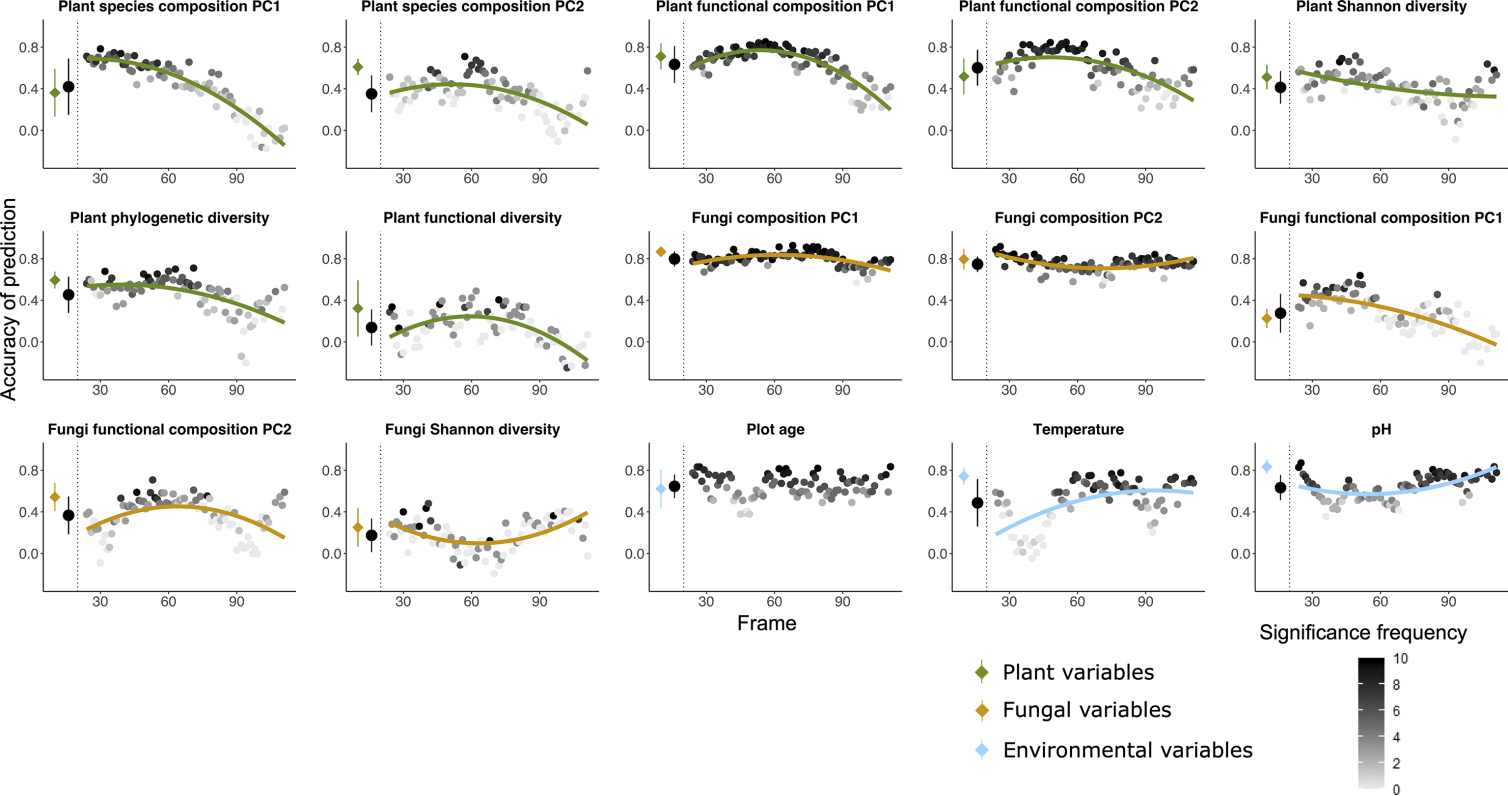
Random forest predictions using the community table of soil bacterial communities (OTU table) as explanatory variable to predict seven variables of plant (green), five variables of fungi (orange) as well as three variables of environmental factors (blue) as dependent variables. The prediction was done both using all the 135 plots and using a moving frame approach. For the moving frame approach, every 45 plots were grouped into one frame and the median plot was used as identifier of the frame. Thus, the first frame included plots 1 to 45, the second 2 to 46, and so forth. The x axis represents the median plot of every frame whose identifiers ranged from plot 23 to plot 113 (i.e. from young successional plots to old plots). The colored circles at the left of each plot denote the mean ± standard deviation of the accuracy of prediction (Pearson’s r) using the full dataset (results of 10-fold cross validation), and the black circles denote the mean ± standard deviation of the accuracy of prediction for all the frames. Each grey to black circle on the right of each plot represents the mean accuracy of prediction of each frame and the color gradient is showing how many correlations of the 10-fold cross-validation were significant with lighter colors indicating less frequent significant predictions. A quadratic or linear regression (the model with higher adjusted *r* ^2^ value) is fit for the gradient if it is significant, showing a change of the accuracy of prediction along the successional gradient.

**Figure 2:**
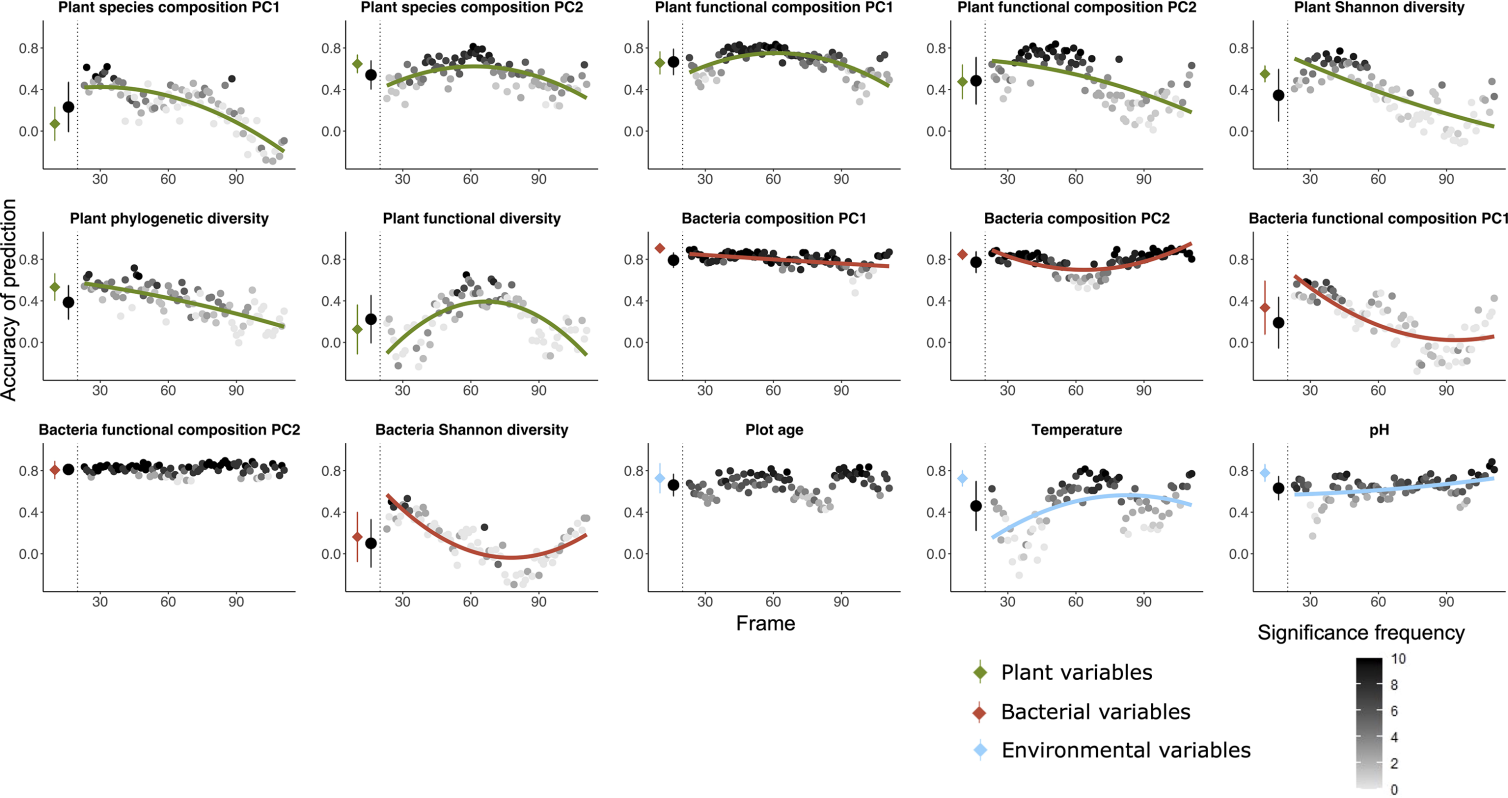
Random forest predictions using the community table of soil fungal communities (OUT table) as explanatory variable to predict seven variables of plant (green), five variables of bacteria (red) as well as three variables of environmental factors (blue) as dependent variables. The prediction was done both using all the 135 plots and using a moving frame approach. For the moving frame approach, every 45 plots were grouped into one frame and the median plot was used as identifier of the frame. Thus, the first frame included plots 1 to 45, the second 2 to 46, and so forth. The x axis represents the median plot of every frame whose identifiers ranged from plot 23 to plot 113 (i.e. from young successional plots to old plots). The colored circles at the left of each plot denote the mean ± standard deviation of the accuracy of prediction (Pearson’s r) using the full dataset (results of 10-fold cross validation), and the black circles denote the mean ± standard deviation of the accuracy of prediction for all the frames. Each grey to black circle on the right of each plot represents the mean accuracy of prediction of each frame and the color gradient is showing how many correlations of the 10-fold cross-validation were significant with lighter colors indicating less frequent significant predictions. A quadratic or linear regression (the model with higher adjusted *r* ^2^ value) is fit for the gradient if it is significant, showing a change of the accuracy of prediction along the successional gradient.

**Figure 3:**
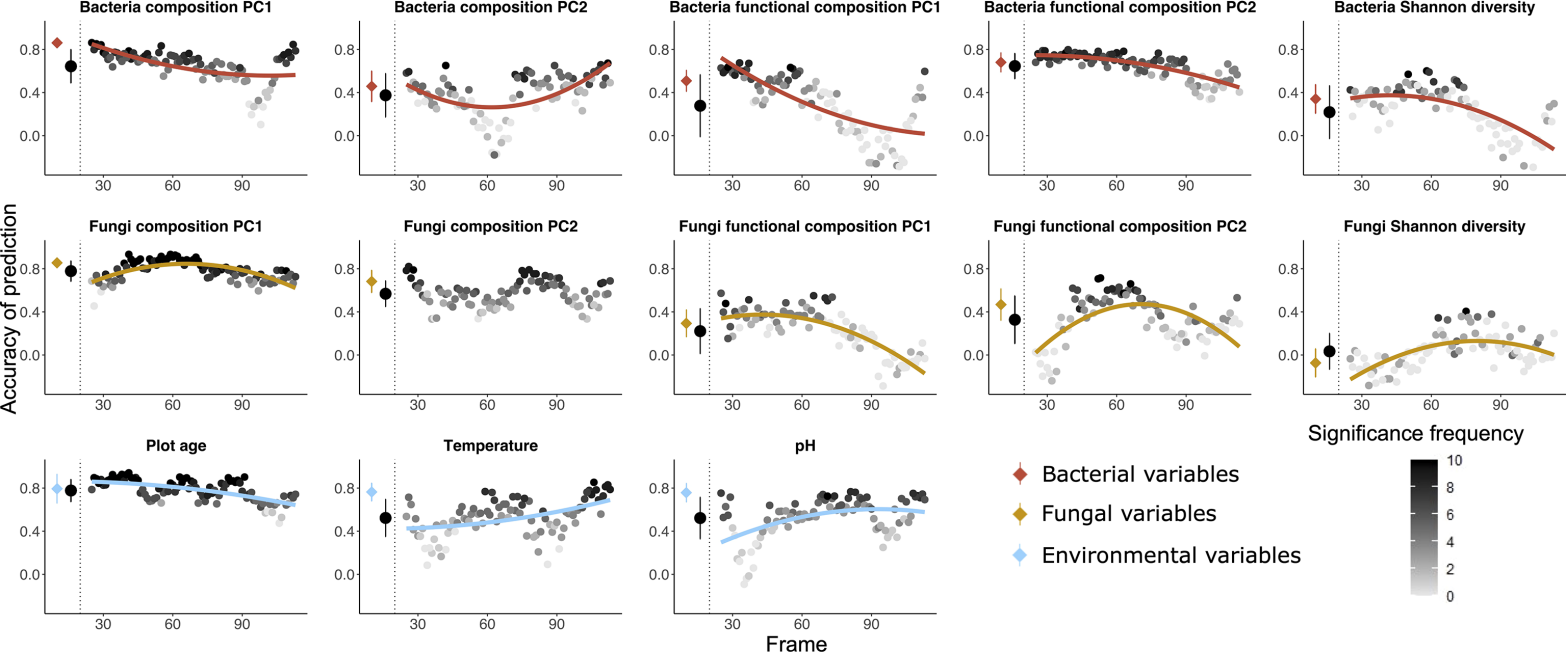
Random forest predictions using the community table of plant communities as explanatory variable to predict five variables of bacteria (red), five variables of fungi (orange) as well as three variables of environmental factors (blue) as dependent variables. The prediction was done both using all the 135 plots and using a moving frame approach. For the moving frame approach, every 45 plots were grouped into one frame and the median plot was used as identifier of the frame. Thus, the first frame included plots 1 to 45, the second 2 to 46, and so forth. The x axis represents the median plot of every frame whose identifiers ranged from plot 23 to plot 113 (i.e. from young successional plots to old plots). The colored circles at the left of each plot denote the mean ± standard deviation of the accuracy of prediction (Pearson’s r) using the full dataset (results of 10-fold cross validation), and the black circles denote the mean ± standard deviation of the accuracy of prediction for all the frames. Each grey to black circle on the right of each plot represents the mean accuracy of prediction of each frame and the color gradient is showing how many correlations of the 10-fold cross-validation were significant with lighter colors indicating less frequent significant predictions. A quadratic or linear regression (the model with higher adjusted *r* ^2^ value) is fit for the gradient if it is significant, showing a change of the accuracy of prediction along the successional gradient.

**Figure 4:**
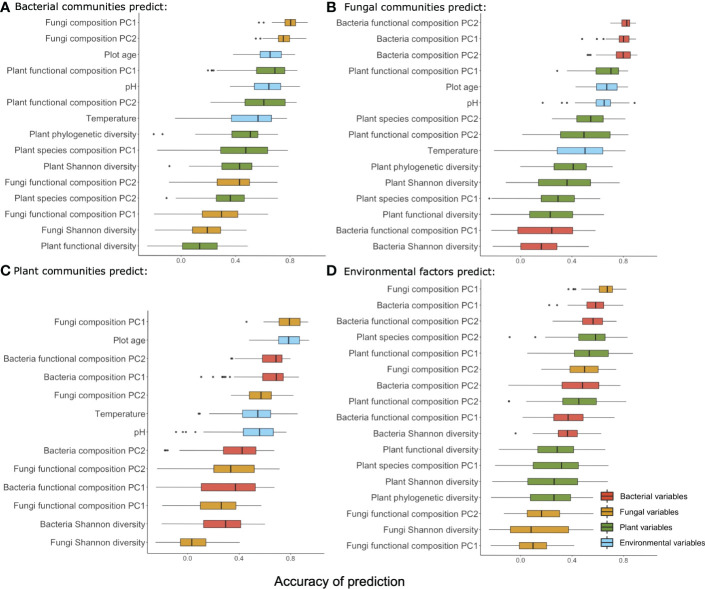
Summary of the accuracy of prediction using taxonomic groups (bacteria **(A)**, fungi **(B)**, plant **(C)**) and environmental factors **(D)** to predict variables from the other three groups along the successional gradient. Variables from each group are color-coded (red: bacteria, orange: fungi, green: plant, blue: environment) and ranked by the mean accuracy of prediction.

In total we obtained soil bacteria and fungi composition data from *n* = 127 and 130 plots after excluding the plots with missing data, respectively; *n* = 4986 bacteria OTUs and *n* = 5701 fungi OTUs were detected in all the soil samples. A total of 99 plant species were identified from 133 plots as plot 1 and plot 6 were unvegetated. Raw sequences of next-generation 16S rRNA gene amplicon sequencing are available at the NCBI Sequence Read Archive (SRA) under the BioProject accession PRJNA701884 and PRJNA701890. The mean accuracy of prediction of each pair of explanatory variables and dependent variables did usually not strongly differ between the global analysis considering all plots and the mean values of the frame-wise analyses, indicating the validity of using moving frames for random forest predictions. Most of the predictions fit a quadratic regression, indicating a non-monotonic change of the accuracy of prediction along the successional gradient (**Figures 1**–**4**).

**Figure 5:**
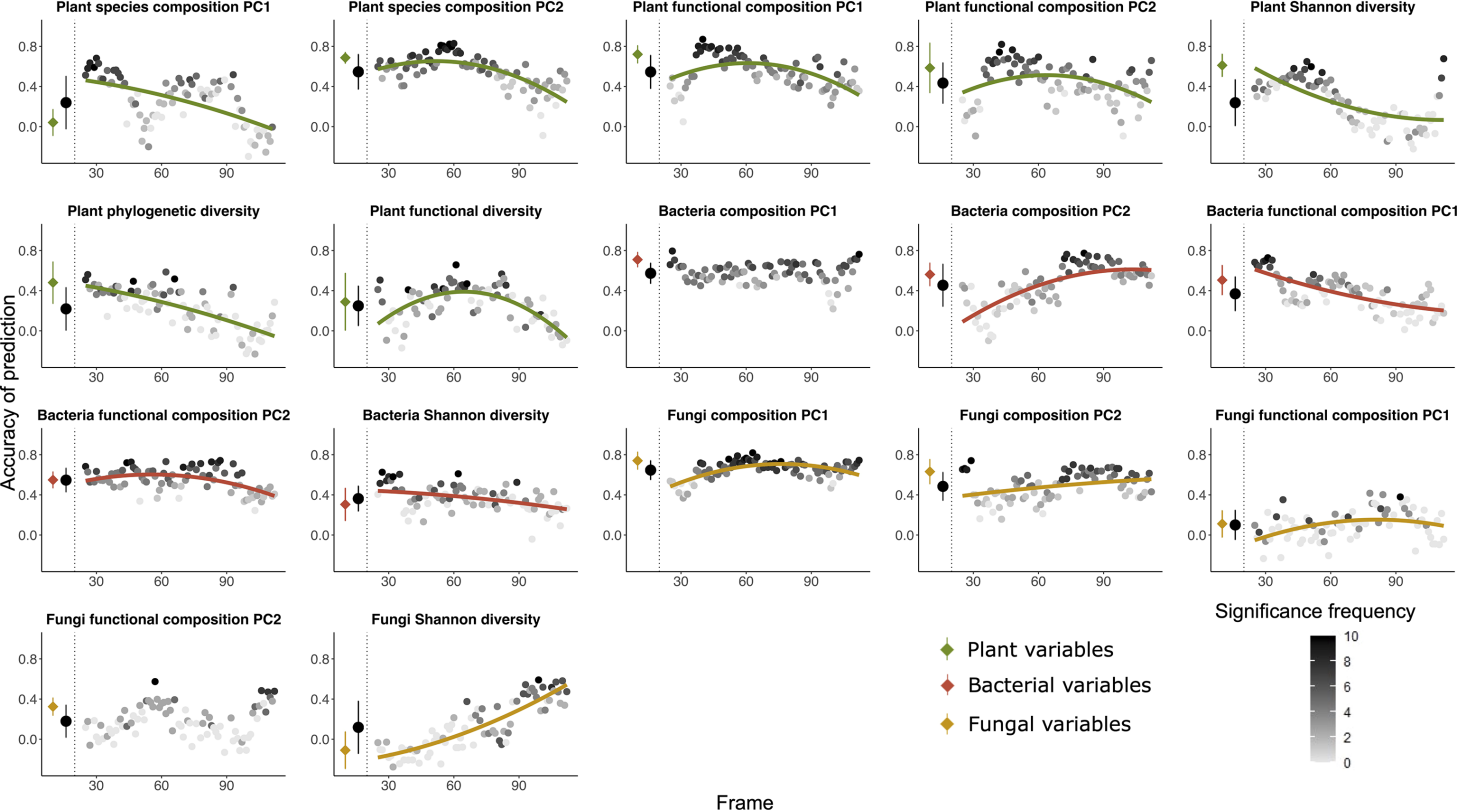
Random forest predictions using all the environmental factors as explanatory variable to predict seven variables of plant (green), five variables of bacteria (red) as well as five variables of fungi (orange) as dependent variables. The prediction was done both using all the 135 plots and using a moving frame approach. For the moving frame approach, every 45 plots were grouped into one frame and the median plot was used as identifier of the frame. Thus, the first frame included plots 1 to 45, the second 2 to 46, and so forth. The x axis represents the median plot of every frame whose identifiers ranged from plot 23 to plot 113 (i.e. from young successional plots to old plots). The colored circles at the left of each plot denote the mean ± standard deviation of the accuracy of prediction (Pearson’s r) using the full dataset (results of 10-fold cross validation), and the black circles denote the mean ± standard deviation of the accuracy of prediction for all the frames. Each grey to black circle on the right of each plot represents the mean accuracy of prediction of each frame and the color gradient is showing how many correlations of the 10-fold cross-validation were significant with lighter colors indicating less frequent significant predictions. A quadratic or linear regression (the model with higher adjusted *r* ^2^ value) is fit for the gradient if it is significant, showing a change of the accuracy of prediction along the successional gradient.

*Bacterial communities as predictors (* **Figures 1**, **5A** *) –* Bacterial communities (quantitative OTU tables) most accurately predicted the taxonomic composition of fungal communities (PC1 and PC2), followed by plant functional composition. Among the environmental parameters, plot age was most accurately predicted by bacterial communities, followed by pH and temperature. Fungi Shannon diversity and plant functional diversity were least accurately predicted among all the variables. Accuracy of prediction by bacterial communities of target variables associated with plant communities mostly decreased with plot age, whereas accuracy of prediction of fungi and most environmental target variables were less variable along the successional gradient or even increased along the age gradient in most cases.

*Fungal communities as predictors (* **Figures 2**, **5B** *) –* Fungal communities (quantitative OTU tables) most accurately predicted the functional composition of bacterial communities (PC2), followed by bacterial taxonomic composition (PC1 and PC2) and plant functional composition (PC1). Plot age was the environmental factor most accurately predicted by fungal communities, followed by pH and temperature. Bacteria Shannon diversity was least accurately predicted among all the variables. Accuracy of prediction of target variables associated with plant communities mostly decreased with plot age, and variables associated with bacterial communities mostly remained constant or decreased, whereas accuracy of prediction of environmental target variables increased along the age gradient in most cases.

*Plant communities as predictors (* **Figures 3**, **5C** *)* – Plant communities (quantitative plant community table) predicted fungi composition (PC1) most accurately, followed by plot age, bacteria functional composition (PC2) and bacteria composition (PC1). Bacteria and fungi Shannon diversity were least accurately predicted among all the variables. The predictions of variables concerning bacteria and fungi were mostly decreasing with increasing plot age. For environmental variables, the accuracy of prediction for temperature and pH increased and for plot age slightly decreased along the successional gradient.

*Environmental factors as predictors (* **Figures 4**, **5D** *)* – Environmental factors (multivariate table of environmental parameters) predicted the fungi composition PC1 and bacteria composition PC1 with the highest accuracy, followed by bacteria functional composition (PC2) and plant species composition (PC2). Fungi Shannon diversity and fungi functional composition (PC1) was least accurately predicted among all the variables. Accuracy of prediction for plant and bacterial variables were often decreasing along the gradient, and for fungal variables they mostly increased with plot age.

## 4 Discussion

Our results showed that the composition and diversity of plant, bacteria, and fungi is overall well predicted by the composition of the respective other taxonomic groups as well as by environmental factors. The accuracy of prediction, however, varied along the successional gradient of the forefield of the Ödenwinkelkees glacier.

Taxonomic groups and environmental factors differed in the ability to accurately predict the composition of the respectively other taxonomic groups. Bacterial and fungal community compositions was the best predictor for each other’s composition, while plant community composition was well predicted both by microbial community composition and environmental factors. In addition, plot age was best associated with plant community composition, followed by fungal and bacterial composition (**Supplementary Figure S3**). This confirms that plant communities represent an ecological succession with age-specific composition, while microbial communities may be predominantly shaped by biotic interactions that are modulated by community age to a lesser extent. In addition, although the PC axes of community composition do not represent the full composition of a taxonomic group, the successful mutual prediction between bacteria and fungi does show that the PC axes are containing a signal of the effects of other taxonomic groups. As stated above, our approach is not implying a direction of effects, which means that it is more likely that the environmental factors affect the composition and diversity of the taxonomic groups and not *vice versa*. Our predictive models may either indicate direct interactions between taxa or common other effects on taxa, as statistical associations between taxa may also suggest that both taxa respond similarly to a third taxonomic group or an environmental factor (Blanchet et al., 2020). In our case, the two environmental factors pH and temperature both have strong associations with all the taxonomic groups after plot age, suggesting their role as important environmental factors defining the community composition of all the taxonomic groups. In addition, temperature was equally well associated with all the taxa and pH had better association with bacterial and fungal composition than with plant composition (**Supplementary Figure S3**), indicating that pH is affecting soil microbes more than plants, which coincides with previous studies illustrating the importance of pH in affecting microbial communities (Knelman et al., 2012; Glassman et al., 2017; Shen et al., 2020). The decrease in soil pH with increasing successional age at our study site confirms similar patterns in other earlier deglaciation chronosequences, where this soil acidification mainly results from increased soil organic matter degradation and the associated leaching of organic acids in late succession (Bernasconi et al., 2011). Compared with the studies conducted in other similar glacier forefields such as Morteratsch glacier and Damma glacier in Switzerland (Burga et al., 2010; Bernasconi et al., 2011), Rotmoosferner glacier in Austria (De Vries et al., 2021), or Hailuogou glacier in China (Jiang et al., 2018), our study aimed at considering the interplay between plants, bacteria, and fungi as well as environmental factors to detect mutual influences in the assembly of multidiverse communities.

Despite the strong associations between pH and temperature with all the taxonomic groups, predictions between the composition of taxonomic groups usually showed better performance than environmental factors. This suggests that apart from the common influence of abiotic factors in affecting the occurrence of different organisms, there is a strong effect of direct interactions between bacteria, fungi and plants which leads to high mutual predictabilities between the taxonomic groups. The importance of biotic interactions in the successional assembly of multidiverse communities is confirmed by previous findings where soil microbial composition was more closely associated to plant communities than to environmental factors (Knelman et al., 2012; Junker et al., 2021). In addition, we found that bacterial and fungal composition serve as better predictors for plant functional composition than for plant species composition, which supports the notion that the functional composition of plant communities strongly impacts its biotic environment (Dassen et al., 2017). Meanwhile, bacterial functional composition was also well predicted by plant and fungi composition, and these together suggest the important role of functional properties for both plant and microbial community.

Predictabilities for biotic and environmental variables varied with successional age. Among all the environmental factors, the associations between pH and temperature with bacterial, fungal and plant community composition changed in a consistent pattern along the successional gradient (**Supplementary Figure S3**). This suggests that in the successional stages where these environmental factors were accurately predicted by all the taxonomic groups, they may have strong positive or negative effects on the occurrence of species of different taxonomic groups, while at the stages where they are not important predictors, other factors may act as the main drivers shaping the communities. Under the dynamic environmental conditions, the mutual predictabilities between plant and microbes at early and late successional stages clearly differed. Plant taxonomic and functional composition were well predicted by bacteria and fungi at early while not at late succession. The change of predictive signal may be caused by the increasing complexity of late successional communities, which may prevent the detection of predictabilities between plants and microbes as noise increases in the data. In our previous study at the same site we found a stronger interdependence between taxonomic groups at late succession (Hanusch et al., 2022), in accordance with an earlier study showing that microbial communities utilize mainly ancient carbon in the first decades after deglaciation while plant-derived carbon becomes a major source for microbes after 50 years of succession (Bardgett et al., 2007). These results suggest that in early successional stages, plants may directly leave a signal of bacterial communities that consume plant derived carbon next to those consuming ancient carbon, which infers the importance of species interactions (Ficetola et al., 2021). In later successional stages, further carbon sources such as decomposed soil organic matters may accumulate, which sustains microbial communities not directly related to plant species diversity and composition, resulting in a decreasing signal of interactions between plants and microbial species pairs with a poor prediction, and this is consistent with the results of glacier succession of Green Lakes Valley in Colorado, USA (Porazinska et al., 2018). Finally, age (inferred from distance to glacier) is not the only factor that is affecting the successional age of plots in glacier forefields, instead allogenic factors may reset successions or at least slow down successional progress in community development (Wojcik et al., 2021). These allogenic factors, such as geomorphic events, accumulate over time and thus may lead to outliers in community composition. If these outlier plots are part of test dataset but not the training dataset, they cannot be predicted on models as predictions are only possible in the range of the training dataset.

Variables describing the composition of taxonomic groups (e.g. PC axis of community composition) were mostly more precisely predicted by other taxonomic groups than diversity indices. Particularly, the community composition of bacteria and fungi mutually predicted each other most precisely, which confirms previous studies demonstrating the interdependences between bacteria and fungi (Miransari, 2011; Deveau et al., 2018). Fungal composition was better predicted by plant composition than bacterial composition, which may reflect the tight interaction between plants and fungi, especially mycorrhiza (Millard & Singh, 2009; Horn et al., 2017; Sweeney et al., 2021). In contrast to the high mutual predictabilities of the composition between the taxonomic groups, the Shannon diversity of all the taxa and the phylogenetic and functional diversity of plants were not well predicted by the composition of other taxonomic groups, suggesting that diversity is not a direct function of community composition. Though plants or microbes may affect the diversity of each other by facilitative or antagonistic effects, the interactions may be regulated by environment and the existence of other taxa as was shown in previous studies (Bennett et al., 2017; Teste et al., 2017; van der Putten, 2017; Raza et al., 2020). In the soil environment multiple bacterial, fungal and plant species interact at the same time with different environmental conditions, and the interplay of these interactions may lead to a hardly predictable complexity of interdependencies and influences between the diversity and composition of different organisms.

Our results demonstrate the concerted development of plants and microbial communities regulated by environmental factors along an alpine glacier chronosequence. We identified how environmental factors define the niches of the organisms at different successional stages and the strongest biotic relationships between taxa in primary succession, revealing the strong interdependencies between taxonomic groups and the dynamic importance of biotic and abiotic factors in shaping natural communities. Our approaches to identify indicators and environmental variables that inform best about the diversity and composition of ecosystems may stimulate the exploration of mechanisms underlying community assembly in future studies, generate hypothesis that can be tested in lab experiments and facilitate monitoring and conservation efforts.

## Data availability statement

The datasets presented in this study can be found in online repositories. The names of the repository/repositories and accession number(s) can be found below: https://www.ncbi.nlm.nih.gov/, PRJNA701884; https://www.ncbi.nlm.nih.gov/, PRJNA701890.

## Author contributions

RJ conceived the study. XH, MH, VR-H, and RJ designed the study and collected the data. XH and RJ analyzed the data. XH and RJ wrote the manuscript with critical input from MH and VR-H. All authors contributed to the manuscript and approved the final version.
