# Supplementary material for: Accuracy of mutual predictions of plant and microbial communities vary along a successional gradient in an alpine glacier forefield
Source: Front Plant Sci. 2023 Jan 13;13:1017847. doi: 10.3389/fpls.2022.1017847 (PMC9880484; doi:10.3389/fpls.2022.1017847)

FIGURES

**Figure S1** The mean value (± standard error) of every frame for the 20 dependent variables (seven variables of plant, five variables of bacteria, five variables of fungi, as well as three variables of environmental factors).


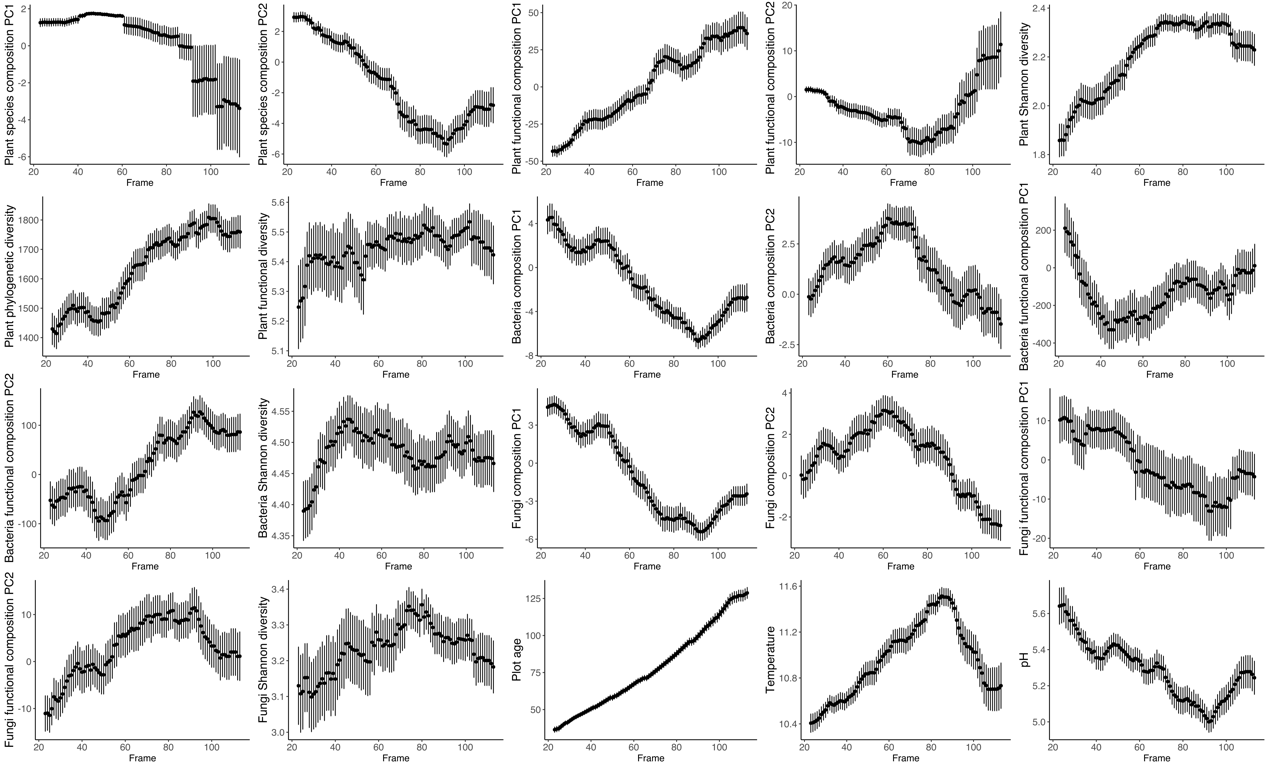


**Figure S2** The correlation graph of the 20 dependent variables (seven variables of plant, three variables of bacteria, three variables of fungi, as well as seven variables of environmental factors).

**Figure S3** Summary of the accuracy of prediction for each variable (seven variables of plant, five variables of bacteria, five variables of fungi, as well as three variables of environmental factors) being predicted by every single group (red: bacteria, orange: fungi, green: plant, blue: environment). The prediction was done both using all the 135 plots and using a moving frame approach. For the moving frame approach, every 45 plots were grouped into one frame and the median plot was used as identifier of the frame. Thus, the first frame included plots 1 to 45, the second 2 to 46, and so forth. The x axis represents the median plot of every frame whose identifiers ranged from plot 23 to plot 113 (i.e. from young successional plots to old plots). Each point represents how well this variable was predicted by other taxonomic groups or environmental factors. The inserted violin plots are the summary distribution for every prediction. The label on each violin is the result of Tukey Test showing if there is significant difference of accuracy of prediction between any pair of predicting groups.


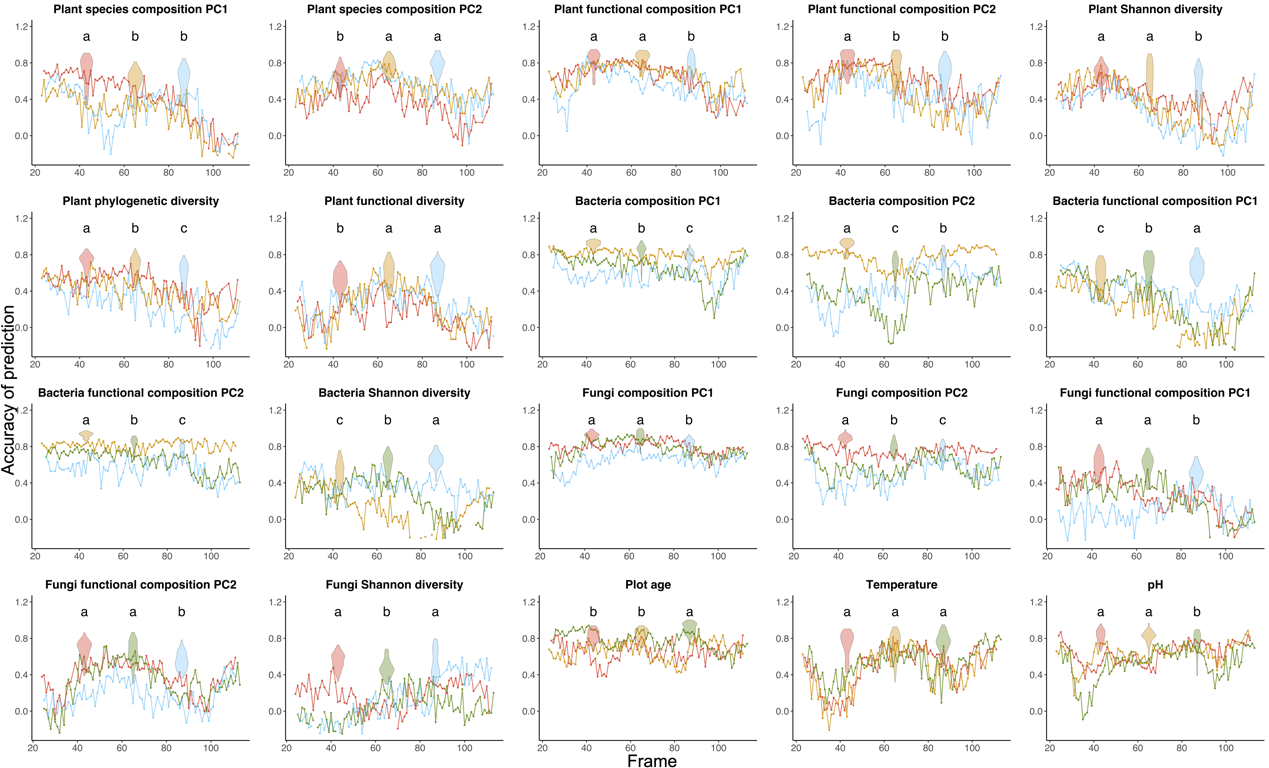

Supplement: Supplementary file 1 [file DataSheet_1.zip › Supplementary Figures.docx]
